# Women Favour Dyadic Relationships, but Men Prefer Clubs: Cross-Cultural Evidence from Social Networking

**DOI:** 10.1371/journal.pone.0118329

**Published:** 2015-03-16

**Authors:** Tamas David-Barrett, Anna Rotkirch, James Carney, Isabel Behncke Izquierdo, Jaimie A. Krems, Dylan Townley, Elinor McDaniell, Anna Byrne-Smith, Robin I. M. Dunbar

**Affiliations:** 1 Department of Experimental Psychology, University of Oxford, South Parks Rd, Oxford, OX1 3UD, United Kingdom; 2 Population Research Institute, Väestöliitto, Kalevankatu 16, 00101, Helsinki, Finland; 3 Department of Social Psychology, Arizona State University, Tempe, AZ, 85287, United States of America; Wenzhou University, CHINA

## Abstract

The ability to create lasting, trust-based friendships makes it possible for humans to form large and coherent groups. The recent literature on the evolution of sociality and on the network dynamics of human societies suggests that large human groups have a layered structure generated by emotionally supported social relationships. There are also gender differences in adult social style which may involve different trade-offs between the quantity and quality of friendships. Although many have suggested that females tend to focus on intimate relations with a few other females, while males build larger, more hierarchical coalitions, the existence of such gender differences is disputed and data from adults is scarce. Here, we present cross-cultural evidence for gender differences in the preference for close friendships. We use a sample of ∼112,000 profile pictures from nine world regions posted on a popular social networking site to show that, in self-selected displays of social relationships, women favour dyadic relations, whereas men favour larger, all-male cliques. These apparently different solutions to quality-quantity trade-offs suggest a universal and fundamental difference in the function of close friendships for the two sexes.

## Introduction

The recent literature on both the evolution of sociality and the network dynamics of human and animal societies [1–8] suggests that large social groups cannot be fully connected: they have a layered structure that is generated by emotionally supported social relationships [9–15]. Although there are structural aspects to social organisation, individual behaviour is crucially shaped by dyadic relationships. It may be that preferred patterns of relationships vary by gender in a way that typically reflects sex differences in reproductive strategies [16–19].

For instance, friendships or close and prolonged affiliation with non-kin are characterised by homophily, so that people typically choose friends of the same age and gender (for recent reviews see [20–22]). Sex differences in reproductive strategies also shape adult social behaviour [20,23–26] and may reflect different trade-offs between the quantity and quality of friendships. Thus, it has been suggested that females invest more heavily in a few, high-quality and time-consuming friendships, while males prefer groups with less investment per member, and higher group cohesion [27–30].

There is reason to believe these gender differences have evolutionary roots. First, sex differences in friendship emerge early [31], are quite apparent already in small children and appear to increase with age [21,32,33]. Second, similar gender patterns exist in some non-human primates [13,34]. One obvious explanation is that male peer sociality evolved to enable hunting, coalitionary support for within-group dominance, and/or defence in larger groups, while women’s peer sociality is to a greater extent shaped by their higher investment in reproduction and child rearing, as well as their historically more frequent experience of out-migration and the need to integrate into a patrilocal society with few, if any, kin [18,20,25]. Patrilocality is also the norm for chimpanzees and bonobos, lending further support to female transfer being the norm throughout hominin evolution [35,36].

However, the scope of human gender differences is disputed. Several studies found few sex differences in the number of close non-related friends that an individual turns to for help and assistance [4,37–42] while others detect crucial differences in the quantity and intensity of male and female peer ties [16,23,24,30,32,43–45]. Sex differences also depend on which component of friendship is being studied, as well as the age and culture of the subjects. For instance, the review by Rose and Rudolph [21] found that girls have a greater preference for extended dyadic interactions and prosocial behaviour, while boys interact more in peer groups with a high network density and clear dominance hierarchy. But gender differences are negligible concerning the expectations males and females have of friends and in the symmetrical reciprocity they expect from them [22]. In any case, documented sex differences tend to be small or medium-size rather than large [22,46].

All reviews stress the need for more friendship research on adults and on people from other than ‘WEIRD’ [47] societies [21,22,46]. Most research on friendships has involved children or teenagers, and there is to date only limited and mixed evidence for gender differences in adult human friendships [20,38,48–50]. Here, we use data from a social media site to explore gender differences in close peer relations. We hypothesised that social relations among same-aged adults would exhibit gender homophily and would vary by gender, such that men would exhibit higher numbers of friends compared to women.

## Methods and Data

To investigate close friendships in the two sexes, we used Facebook Profile Pictures following the example of recent literature that deals with social networking data [51–54]. Facebook is the most popular global social networking site, the primary function of which is to fulfil psychosocial needs for belonging and self-presentation [55]. Upon signing up, each user may choose a Profile Picture, which represents the user to the rest of the Facebook community, is public, and appears at the top of the user’s profile and as the icon next to the user’s name wherever he or she posts on the site. Each user can have only one Profile Picture at a time. Usually the picture features the user only. When the picture displays peers they tend to be the user with friends or acquaintances [51]. The choice of profile pictures is related to the user’s desired impression formation [55]. This impression is not, however, too detached from the real world: Facebook user profiles have been found to reflect actual rather than idealised identities [56]. We thus assume that Profile photographs of peers are likely to align with behavioural inclinations, and thus provide a reliable proxy for relationship preferences.

### Data collection

We used random search terms to select 309 users (seeds) of Facebook who shared their batch of friends publicly. (The 309 users had on average 362 friends.) In the first wave of data collection, we located the Profile Pictures of all the friends of each of these users (111,863 Profile Pictures in total); each photograph was categorised with respect to the type of the picture, and the number and gender of the persons displayed (see Table 1). Data collection took place between July 2011 and January 2012.

**Table 1:** Coding categories.

Code	Description	Tally	%
NH	Not human picture: e.g., object, landscape, monster, car, or any picture with an animal	13,861	12.9
NA	Not publicly available (or Facebook default profile)	1,626	1.5
NP	Multiple people but not peer: e.g., mother-child, a family	2,651	2.5
CB	Child or baby	3,470	3.2
MP	Multiple pictures, collage	1,961	1.8
CTG	Can’t tell gender	2,447	2.3
1F	1 female	32,208	30
2F	2 females	3,508	3.3
3F	3 females	945	0.9
4F	4 females	384	0.4
1M	1 male	30,279	28.2
2M	2 males	2,326	2.2
3M	3 males	935	0.9
4M	4 males	388	0.4
1F1M	1 female + 1 male (e.g. a couple)	6,769	6.3
1F2M	1 female + 2 males	329	0.3
2F1M	2 females + 1 male	359	0.3
1F3M	1 female + 3 males	106	0.1
2F2M	2 females + 2 males (e.g. two couples)	185	0.2
3F1M	3 females + 1 male	110	0.1
5+	Five or more people on the picture	2,433	2.3

Additional information was collected in two further waves. In the first of these (wave 2), geographical data based on geographical location was collected to control for cultural differences. The coders were provided with a list of geographic regions: Europe (56 seeds), Central and South Asia (16), Latin America (9), Middle East and North Africa (14), North America (14), South East Asia (43), Sub-Saharan Africa (7), Australia (9), East Asia (19), or ‘can’t tell region’ (21). In the second (wave 3), we used a finer classification of the number of people shown in Profile Pictures: the first two waves classified all pictures with four or more individuals as a single category, but in wave 3 this was extended to specify individual numbers up to 20.

The coding was done by 8 coders, all but two of whom were research assistants at the University of Oxford. All coders bar one (the lead author) were blind during the coding phase, knowing only that the project was to study gender differences on social networking sites. Coders were instructed to avoid any discussion of the project amongst themselves during the coding phase. All results presented in this paper are robust to the elimination of each of the coders, each of the regions, and the type of Profile Pictures displayed by the seeds.

We used only publicly available pictures. No pictures or other information associated with this research was either separately downloaded or stored. The research project was approved by the Central University Research Ethics Committee of University of Oxford, and each coder received a full research ethics briefing before joining the coding team.

### Data exclusion

**Fig 1:**
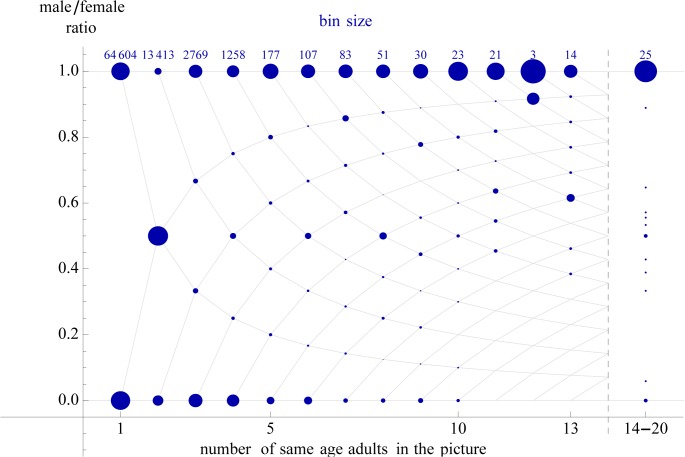
The ratio of men in Profile Pictures (with same age adults only) as a function the number of persons in the picture. The value corresponding to each *n* adds up to 1. The size of the disks denotes the share in the ratio at that particular bin. Crossing points on the grid are the only possible points given the discrete nature of the data.

As adult interpersonal processes were our focus here, we excluded all Profile Pictures that contained a non-human figure, a baby or child, people of markedly different ages (as judged by the coders), pictorial collages (i.e. pictures composed of multiple, distinct or coloured photos), a person or people whose gender(s) were unidentifiable or that did not contain a person. 81,246 Profile Pictures remained, of which 19,984 (26%) contained more than one person. For an overview of the data see Fig. 1.

## Results

### Gender homophily

First, we studied gender homophily in profile pictures. As expected, if the Profile Pictures displayed three or more persons (7.6% of Profile Pictures displaying adults), they tended to be the same gender, confirming our gender homophily hypothesis (Fig. 1).

**Fig 2:**
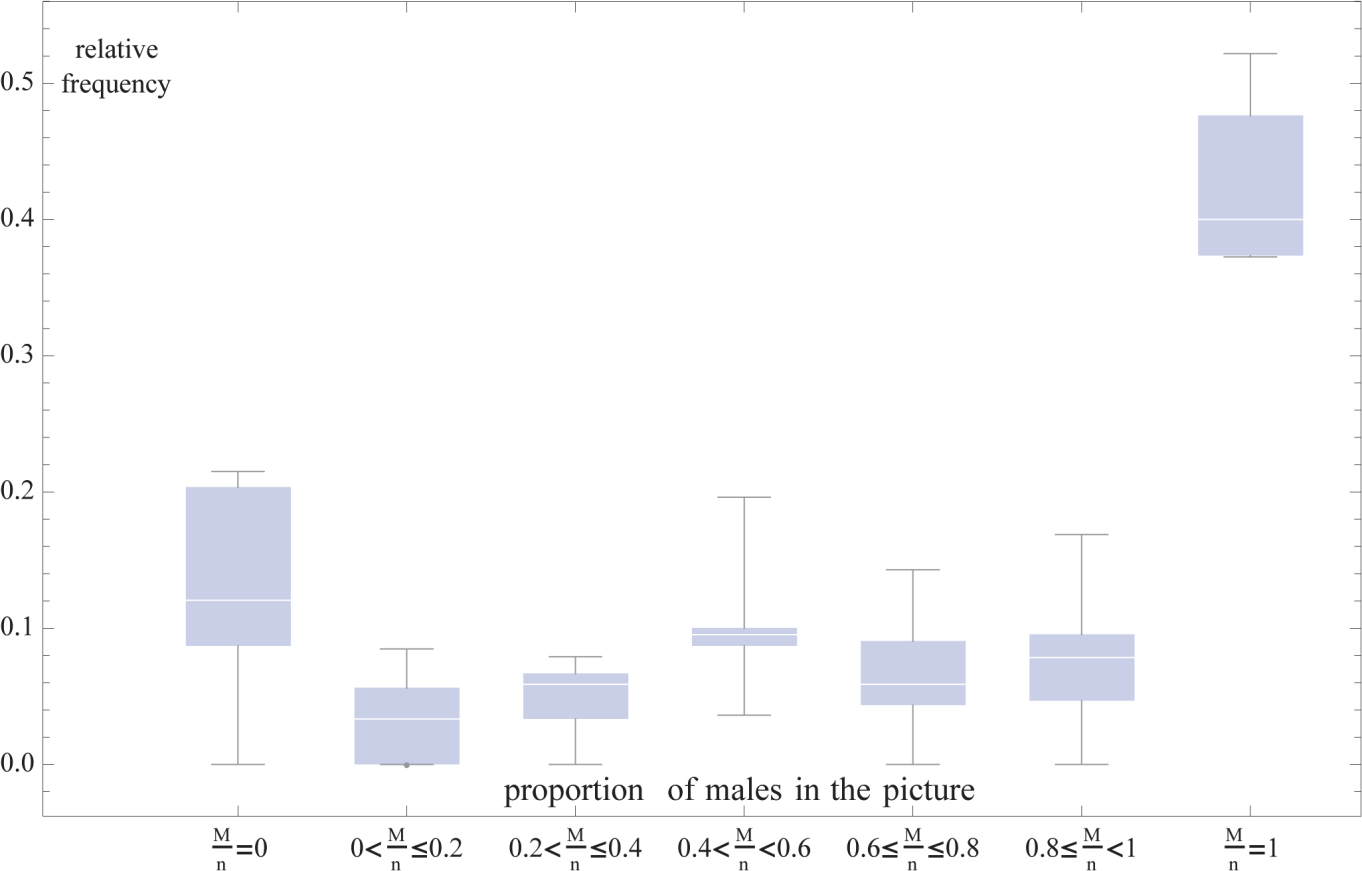
Relative frequencies of different gender ratios for groups where 5< = n< = 12. The distribution shows a trimodal pattern with women-only, gender-equal and men-only gender ratios significantly above zero.

The gender ratio on Profile Pictures persons exhibited a tri-modal distribution (see Fig. 2). In Profile Pictures with 5–12 persons, the frequencies for women-only, men-only, and equal gender ratios are 15.5%, 40.6%, 15.7%. (Note that for *n*<5 the trimodality cannot exist, while for very large *n* the male groups dominate to such an extent that only one modality is left.) These are above the frequencies for all other combinations: pictures with both genders but unequally represented (such as one woman and two men) were significantly less common. Given that the population mean is approximately balanced, this finding suggests a strong gender homophily.

**Fig 3:**
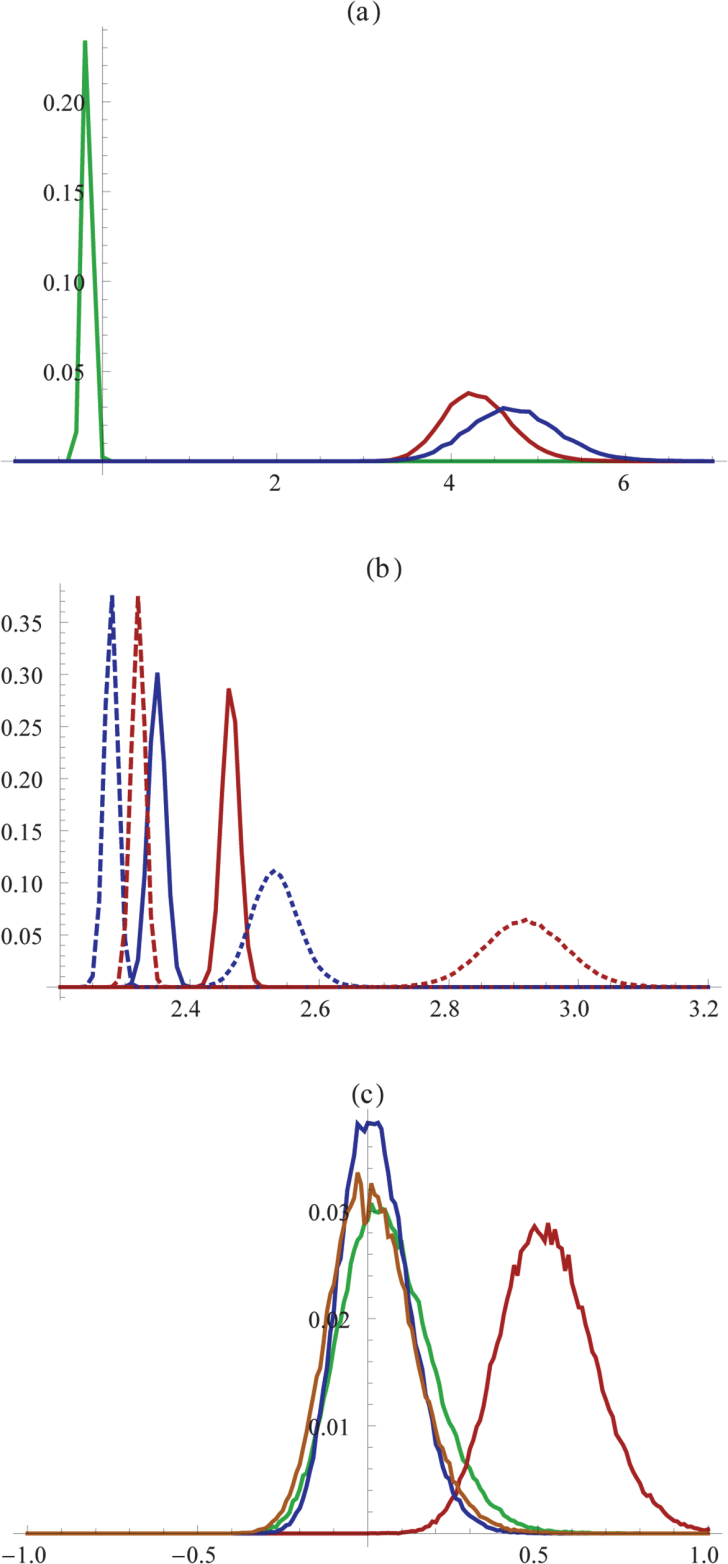
Bootstrap histograms. Panel (a): the ratio of same gender Profile Pictures compared to mixed gender profile pictures (probability corrected); green, red, and blue lines correspond to n = 2, 3, and 4. Panel (b): the number of people on a Profile Picture men (red) or women (blue) appear on; straight lines: same gender with *n* between 2 and 4, dashed lines: mixed gender with *n* between 2 and 4, dotted lines: mixed gender with *n* between 2 and 20. Panel (c): the ratio of the frequency of same-gender pictures between the genders; green: pictures with 1 person, red: 2 persons, brown: 3 persons, and blue: 4 persons. (100,000 bootstrapping repeats.)

This preference for same-sex friends was further supported by the subsample that contains Profile Pictures displaying fewer than 5 people. While pictures with two persons showed a higher mixed than same gender frequency (sample average (2F+2M)/1F1M-1 = -0.123 (bootstrapping mean -0.122 and s.d. 0.044), likely indicative of pictures of romantic couples, both the 3-person pictures (sample average 2(3F+3M)/(1F2M+2F1M)-1 = 4.34 (bootstrapping mean 4.36 and s.d. 0.39) and the 4-person pictures (sample average 3(4F+4M)/(1F3M+2F2M+3F1M)-1 = 4.78 (bootstrapping mean 4.75 and s.d. 0.51) had a much higher same-gender frequency compared to mixed-gender frequency (Fig. 3A).

### Male propensity towards displaying more people

**Fig 4:**
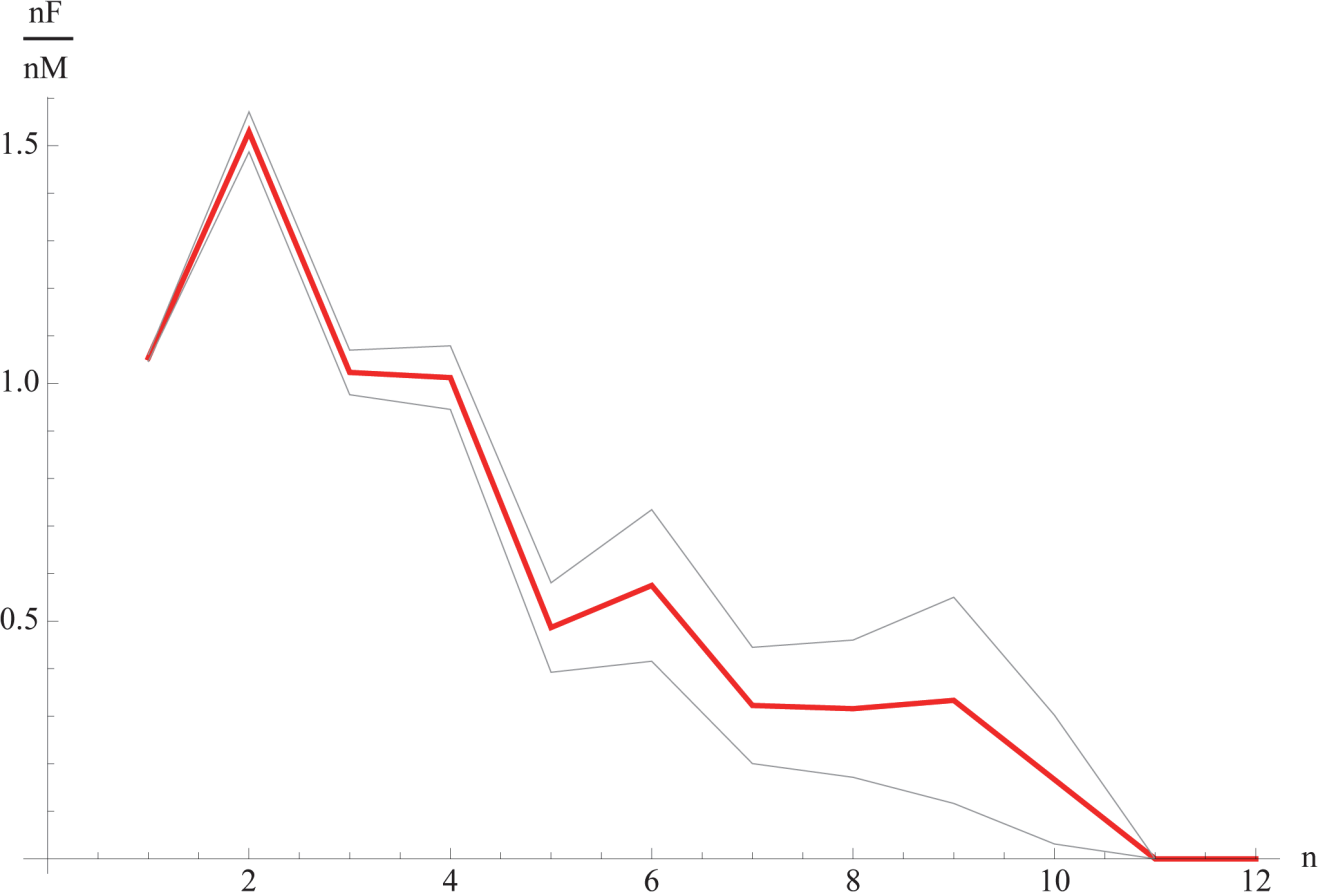
The ratio of same-gender women-only to men-only frequencies (nF/nM as function of n). Gray lines denote a one-standard-deviation band from bootstrapping. Above n>4, men-only groups dominate, while women-only groups become extremely rare. The linear OLS coefficient of nF/nM as a function of n is negative, with R^2^ = 0.86, and p<0.01

Studying the gender composition of peer groups indicated that, when Profile Pictures display a large group of people, they tend to be all male, which was in line with our expectations. In groups of three or four persons, men and women had the same propensity to appear in same-gender Profile Pictures (Table 1). However, larger groups were predominantly male, and increasingly so as the group size grows (Fig. 4).

The male propensity to be part of larger groups was further supported by the fact that men appear together with more people than do women (Fig. 3B). For groups with 2–4 peers of the same gender, men appeared in pictures with 2.47 people on (bootstrapping mean 2.47, s.d. 0.01), as opposed to 2.35 of women (bootstrapping mean 2.35, s.d. 0.01), which are significantly different from each other (p<0.0001). For groups with 2–20 peers of any gender, men appeared with 2.90 (bootstrapping mean 2.90, s.d. 0.07), as opposed to 2.54 of women (bootstrapping mean 2.54, s.d. 0.05) which are also significantly different from each other (p<<0.0001). This suggests that independently of gender combinations and group size, men tend to appear with a larger number of people displayed in Profile Pictures.

### Women focus on dyadic relationships

Finally, we detected an unexpectedly strong female focus on same gender dyads. Women not only tended to appear in Profile Pictures displaying a smaller number of people, as expected in the second research hypothesis, but there was also a strong preference towards pictures containing two women (Fig. 3C). There were 50.8% more pictures with two female peers than pictures with two male peers (bootstrapping mean 51.5, s.d. 14.8). This is especially remarkable given that same-gender pictures with 1, 3, or 4 or more people had an almost perfect gender balance: there were only 6.4% more pictures with one woman only in our dataset than with one man only (bootstrapping mean 7.2, with s.d. 14.1), only 1.1% more pictures with three women than with three men (bootstrapping mean 1.7, s.d. 10.8), and 1.0% fewer pictures with four women compared to four men (bootstrapping mean 0.5, s.d. 12.5).

### Cultural variation

**Fig 5:**
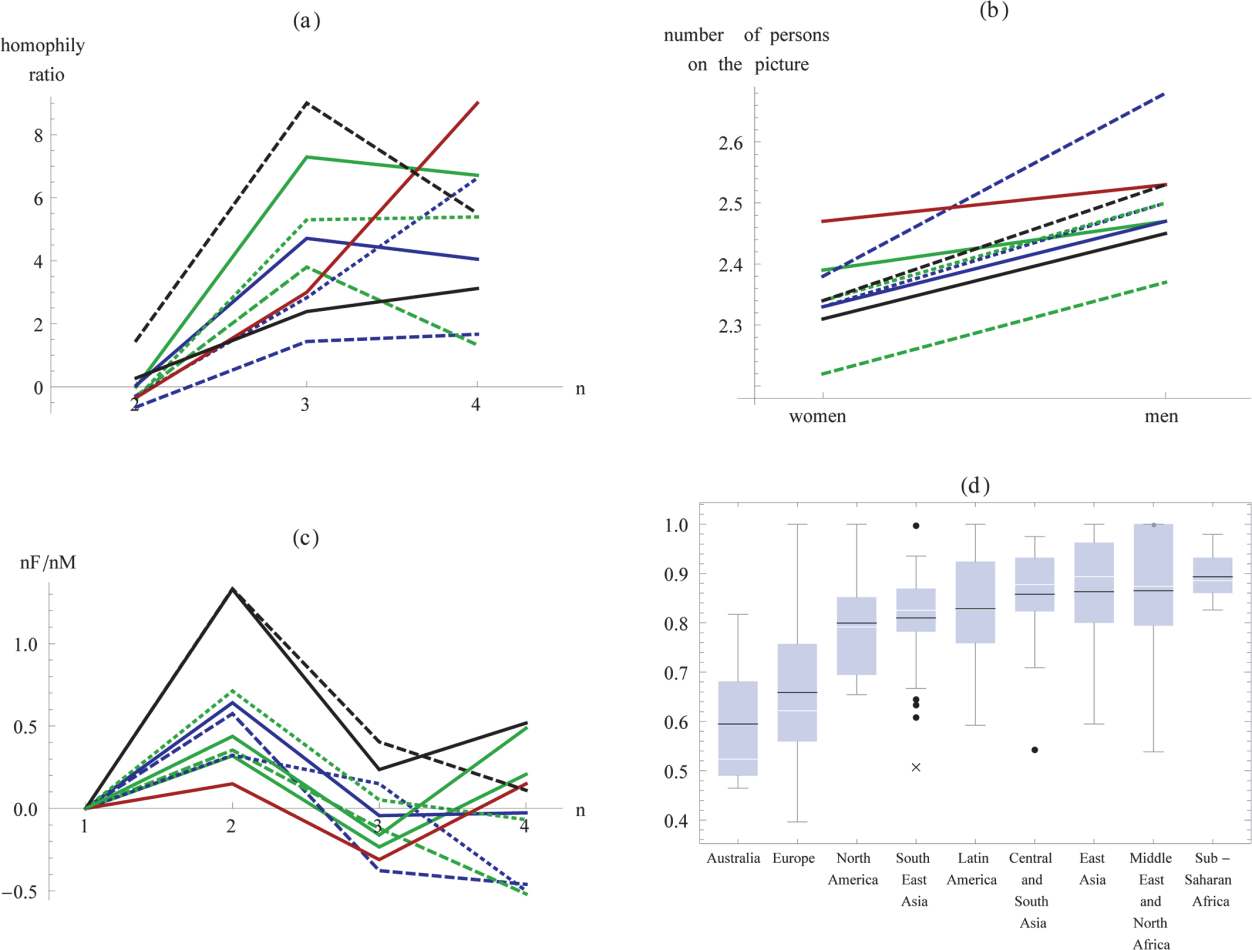
Cultural variation in the main finding. Panel (a): the ratio between same-gender and mixed gender pictures of different group size (corrected by the probability of appearance, see text). Panel (b): number of close friends (same gender, with groups size 2 to 4). Panel (c): the ratio between same-gender Profile Pictures as a function of group size (1F/1M normalised to 0). Panel (d): cultural variation in the proportion of single person pictures within all Profile Pictures in a given global region. (Region codes of Panels a-c: green: Central and South Asia, blue: Europe, dashed blue: Latin America, dashed green: Middle East and North Africa, dotted blue: North America, dotted green: South-East Asia, red: Sub-Saharan Africa, black: Australia, dashed black: East Asia.)

Although there was substantial variation across the different world regions, the main patterns of our findings were present in each of them(Fig. 5A-C), with only the magnitude of the effect varying. While a selection bias may theoretically have affected the relatively small local variation of our results, we find this unlikely: the seeds were randomly selected and at the time of the data collection 17% of the global adult population was using Facebook. Furthermore, measures for other than our three main findings reported above were less uniform among the world regions (Fig. 5D).

## Follow-Up Studies

It is possible that the relative prevalence of female-female Profile Pictures in comparison to male-male ones may not reflect friendship behaviour but either (a) a female preference for putting up pictures of two people of any gender, or (b) a reluctance among men to display pictures with two male friends, especially in regions where homophobia is common. We tested both of these alternative explanations.

First, we tested whether there is a gender difference in the preference for Profile Pictures containing two people only. We randomly selected 960 new profiles on Facebook with two same-aged individuals on them. Out of the 960, we were unable to determine the gender of the account user in 11 cases. Out of the remaining 949 cases, 493 pictures belonged to a man and 456 belonged to a woman. Given the fact that the overall Facebook participation gender ratio is almost balanced, this result suggests that the probability of women having a strong preference to put up pictures of two people of *any* gender is negligible.

**Fig 6:**
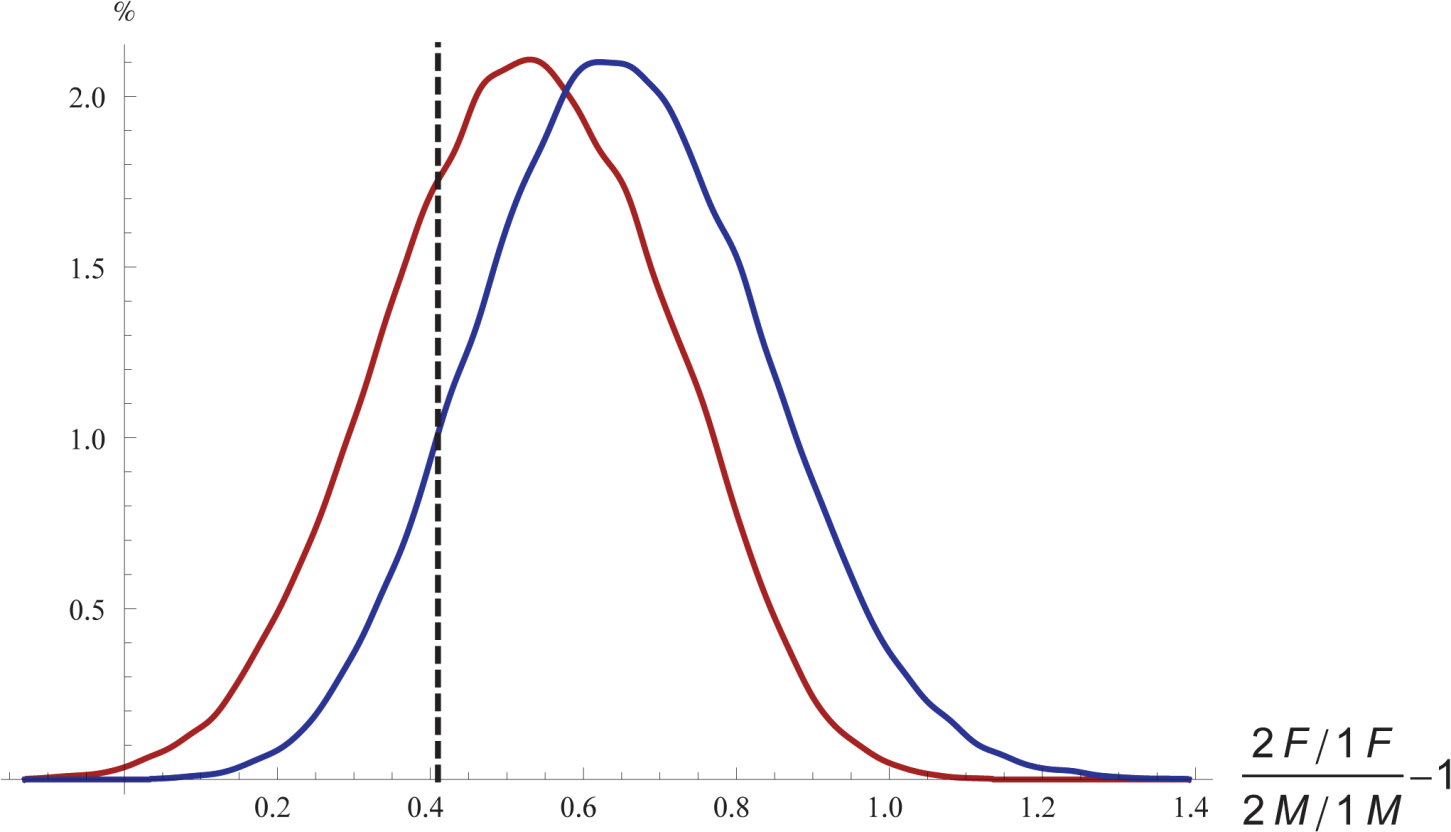
Homophobia is not related to the frequency of 2F Profile Pictures, bootstrapping distributions. Blue line: highly homophobic countries; red line: countries with low homophobia (see text for definitions). Dashed black line: the (2F/1F)/(2M/1M)-1 mean of the entire database. (100,000 bootstrapping repeats.)

Second, we tested if homophobia in the country where the seed owner of the Facebook account lived would affect the ratio between female and male same-gender two-person pictures. (To correct for the fact that different countries have different gender ratios among Facebook users, we used the (2F/1F)/(2M/1M)-1 measure.) As a measure of homophobia, we used the country level geographical codes of our data, and the corresponding homophobia index for these countries as calculated by Pew Global [57]. As the homophobia index of Pew Global covers only the largest 52 countries, we could not test this hypothesis on our entire database. For the remaining seeds, countries were coded to be low on homophobia if they scored between 0 and 40 for the question “Homosexuality is a way of life that should be accepted by society” and to be high homophobic countries if they scored between 60 and 100. This gave us 66 seeds living in countries with low homophobia and 47 seeds in countries with high homophobia. The (2F/1F)/(2M/1M)-1 mean for the two groups were 0.63 and 0.52, respectively (Fig. 6). As the difference is not statistically significant (p = 0.12), and is in the opposite direction to that predicted by the hypothesis, we can conclude that male homophobia is not associated with the difference between 2F vs 2M frequencies in our dataset.

## Discussion

Human societies are complex, large-scale communities of multi-generational social networks. At base, however, these networks are built of the small-scale personal networks of individuals. Our data shed light on the dynamics of these personal networks by providing strong cross-cultural evidence for the universality of a male propensity to prefer a higher number friendships compared to women. While large women-only groups were almost non-existent in self-selected Profile Pictures, males were more likely to present themselves as part of large all-male groups—arguably an essential element of male-male coalitional competition. Our results are broadly in line with many previous studies of human friendship [27,30,58,59] which also found strong gender homophily and male preference for coalitions. This difference in the preferred number of friends may signal different solutions to the quantity-quality trade-off in social ties. The emotional quality of a relationship is a positive function of the time invested in it [39,60], and the closer and more time-demanding a relationship one has, the less time can be devoted to others [11,24]. At the same time, the amount of social capital available that individuals have to distribute among the members of their personal social networks is limited [61,62]. It thus appears as if women build a ‘dense’ network, while men make alliances based on ‘loose’ networks. We also found gender similarity in the preferences for three and four friends, which may explain part of the inconclusive results in previous studies on sex differences in friendship numbers.

The male propensity to form coalitions could have emerged from the sexual division of labour in ancestral environments. It was a male responsibility to defend the group against attack from outsiders, and to do so successfully it was necessary that men band together [20,24]. In males but not females, then, out-group defence called for coalitional cooperation and behaviour.

Our finding that women prefer to picture themselves with fewer friends, and thus appear more often to focus their social capital on only one person at a time, suggests a strong female preference for dyadic relations. The social benefits of such a female dyadic social style are harder to pin down, but three alternative hypotheses might be suggested. First, females may have developed a propensity to form dyadic same-sex friendships as a response to the challenges of their social environments. Given the likelihood of ancestral patrilocality [35,36,63,64], adolescent females would often have entered communities where they had few or no close kin. For females especially, the presence of kin is fitness-enhancing, as has been repeatedly shown for both anthropoid primates [65,66] and humans [67,68]. Thus the formation of emotionally intense, exclusive and “sisterly” dyadic bonds may have been a means to essentially replace kin [25] and to defend against male and inter-female aggression in the new community where she did not have female kin [25,69,70]. This mirrors the case of patrilocal bonobos (*Pan paniscus*), where females enter foreign communities in adolescence and integrate into their new group through intense bond formation with another (typically older) female [71]. A second explanation posits that, since females are the driving agents in human pair-bond formation, it may be a female-specific sexual strategy to form exclusive dyadic relationships. In this framework, the high frequency of female-female dyads in women’s lives might be a by-product of a preference for pairbonding [12]. A third explanation focuses on females’ unique capacity for intense empathic relationships, derived from the mother-infant bond. In this model, heightened female empathy creates an emphasis on individual relationships as a consequence of the psychological toolbox of mothering [72,73]. In comparison, males generally neither have nor require this capacity, and hence they form less emotionally close bonds, those of friendship included.

These three explanations for gender differences in social style—patrilocality, pair-bonding and maternal empathy—are difficult to tease apart. Not only is the evolutionary origin of all primate bonding likely to have arisen out of the mother-infant relationship [74], whatever forces shaped female friendship thereafter, different ultimate causes (e.g., defence against aggression in a patrilocal society, or assistance among maternal kin) may have used similar proximate mechanisms (e.g., high reliance on intimate disclosure) making it hard to dissociate them. Furthermore, recent studies of close friendship as a function of age suggests that women switch their primary focus from female-female friendships to pairbonding and then to mother-daughter bonding at different stages of the life cycle [75].

The importance for a female of maintaining close relationships once she has left her natal group sheds light on the strategies that women use during intrasexual aggression (notably exclusion and relationship-ending gossip [29,69]). If a female’s bonds to friends and her spouse are crucial for accessing resources—from food to information—then breaking these bonds and/or excluding the female all together can radically affect that individual’s fitness, to the benefit of her competitors.

There are, inevitably, some potential limitations to our data. We cannot be sure that co-appearance on Profile Pictures always reflects real-life social ties. Future research is needed in order to assess gender differences in offline sociality. However, no existing research suggests that profile pictures would include imagined or random social relations to any significant extent (not least because the other person is likely to object) and our results are in line with other recent findings from online social communities [50]. Displaying Profile Pictures with two or more people compared to only one person may also reflect some unknown personal psychological characteristics or specific life-events of account users; however, such possible characteristics should not affect our main results.

In summary, our results point to striking gender differences in intimate friendship strategies: women prefer close dyadic bonds (with evolutionary origins in either pairbonding or social insurance purposes, or both), whereas men use their bonding capacity to build multimale groups (in effect, clubs). This concurs with work on chimpanzees, where females form tight, kin-based networks and males make loose, easy-to-break alliances [76]. Since similar gendered bonds are found in our closest primate relatives, they may long predate the evolution of our species. Among cercopithecine primates, females are disproportionately more likely to invest in core female ‘friendships’ with matrilineal relatives as group size gets larger, apparently in order to maximise the effectiveness with which these relationships function as social buffers [1,12,65]. By contrast, chimpanzees [34] and humans may show a tendency to form close friendships with unrelated females in addition to those they might form with close female relatives.

## Supporting Information

S1 Data Anonymised profile picture frequency database. (CSV)
